# Supplementary material for: mRNA-1273 is placenta-permeable and immunogenic in the fetus
Source: Mol Ther Nucleic Acids. 2025 Feb 17;36(1):102489. doi: 10.1016/j.omtn.2025.102489 (PMC11919431; doi:10.1016/j.omtn.2025.102489)
Supplement: Document S1. Figure S1 [file mmc1.pdf]

OMTN, Volume *36*

Supplemental information

mRNA-1273 is placenta-permeable and immunogenic in the fetus

Jeng-Chang Chen, Mei-Hua Hsu, Rei-Lin Kuo, Li-Ting Wang, Ming-Ling Kuo, Li-Yun Tseng, Hsueh-Ling Chang, and Cheng-Hsun Chiu

A

Derivation of the mathematical formula for mRNA quantification

In reverse transcription, **[cDNA] = [mRNA] x Yield (%)**

In quantitative PCR, **[DNA amplicons] = [cDNA] x 2^{Ct} = [mRNA] x Yield x 2^{Ct}**

⇒ **[DNA amplicons] / Yield = [mRNA] x 2^{Ct}**

Take natural logarithm (Ln) of both sides

Ln([DNA amplicons] / Yield) = Ln([mRNA] x 2^{Ct})

= Ln[mRNA] + Ln(2^{Ct})

= Ln[mRNA] + Ct x Ln(2)

= Ln[mRNA] + Ct x 0.6931

So **Ln[mRNA] = - 0.6931 x Ct + Ln([DNA amplicons] / Yield)**

In the case of common (decimal) logarithm, the formula will be:

log[mRNA] = - 0.3010 x Ct + log([DNA amplicons] / Yield)

- **[mRNA]** is the amount of sample mRNA that serves as the template to reversely transcribe cDNA, recorded as **[cDNA]**.
- **Yield** refers to reverse transcription efficiency for the reverse transcriptase used.
- **[DNA amplicons]** represents **[cDNA]** products after amplification for a period of **Ct**.
- **Ct** is the cycle threshold, as the thermal cycles required for the fluorescent signal to cross the threshold (corresponding to **[DNA amplicons]**) in the exponential phase of DNA amplification.

B

3-fold serial dilutions of cDNA

Ct values	Moderna CmRNA (pg/uL)	Ln(CmRNA)
6.4105	10,000.0000	9.2103
9.9689	3,333.3333	8.1117
14.7061	1,111.1111	7.0131
16.2043	370.3704	5.9145
17.8025	123.4568	4.8159
19.3628	41.1523	3.7173
21.0108	13.7174	2.6187
22.7968	4.5725	1.5201
24.7587	1.5242	0.4214
26.2916	0.5081	-0.6772
27.6568	0.1694	-1.7758
29.3245	0.0565	-2.8744
31.0932	0.0188	-3.9730
32.9435	0.0063	-5.0716
34.8456	0.0021	-6.1702

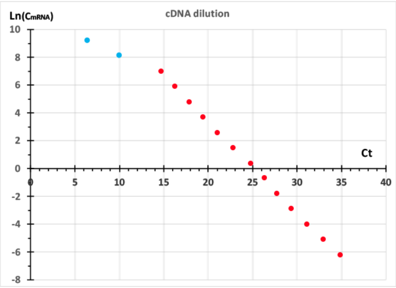

3-fold serial dilutions of mRNA

Ct values	Moderna CmRNA (pg/uL)	Ln(CmRNA)
5.3557	10,000.0000	9.2103
5.9440	3,333.3333	8.1117
10.8954	1,111.1111	7.0131
12.8743	370.3704	5.9145
14.9249	123.4568	4.8159
16.5962	41.1523	3.7173
17.9110	13.7174	2.6187
19.7502	4.5725	1.5201
21.6020	1.5242	0.4214
23.4505	0.5081	-0.6772
24.7337	0.1694	-1.7758
25.9041	0.0565	-2.8744
25.8494	0.0188	-3.9730
27.8912	0.0063	-5.0716
26.6382	0.0021	-6.1702

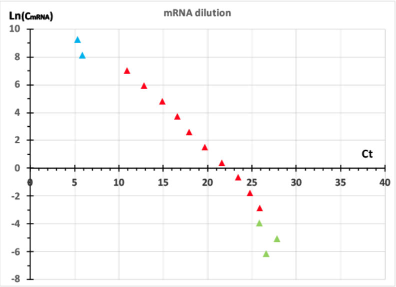

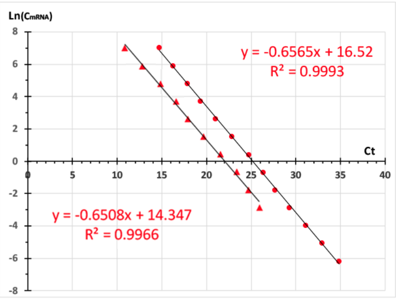

C

Ct values	Moderna CmRNA (pg/uL)	Ln(CmRNA)
5.6808	10,000.0000	9.2103
7.7765	3,333.3333	8.1117
12.5616	1,111.1111	7.0131
14.2327	370.3704	5.9145
15.8015	123.4568	4.8159
17.2555	41.1523	3.7173
19.1251	13.7174	2.6187
21.0317	4.5725	1.5201
22.7946	1.5242	0.4214
24.1883	0.5081	-0.6772
25.7385	0.1694	-1.7758
27.2565	0.0565	-2.8744
28.6493	0.0188	-3.9730
30.1717	0.0063	-5.0716
32.0168	0.0021	-6.1702
33.1922	0.0000	Negative control

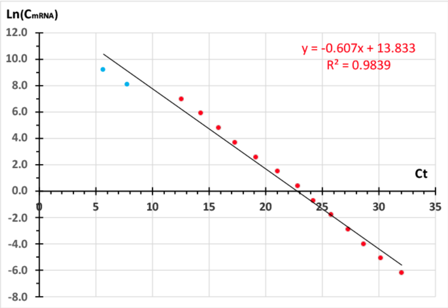

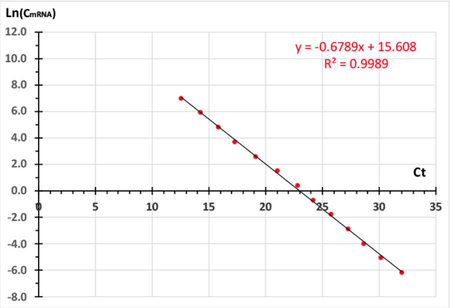

Figure S1. Absolute quantification of spike mRNA by RT-PCR (A) The formula for spike mRNA quantification was mathematically derived. In reverse transcription, cDNA transcripts are converted proportionally to input target mRNA amount,[S1,2] shown as [cDNA] = [mRNA] x Yield (%).[S3] Yield refers to reverse transcription efficiency for the reverse transcriptase used. In quantitative RT-PCR using cDNA reversely transcribed from mRNA, Ln[mRNA] is linearly related to Ct with a slope of -0.6931 and a vertical intercept of Ln([DNA amplicons] / Yield). **(B)** Serial dilution strategies of spike mRNA influenced the detection sensitivity of the spike mRNA standard curves. The standard curve of spike mRNA in RT-PCR was created in

reference to mRNA-1273, using 3-fold serial dilutions of cDNA reversely-transcribed from 10^4 pg/μL mRNA-1273 (circular data points, upper panels) or cDNA reversely-transcribed individually from 3-fold serial dilutions of 10^4 pg/μL mRNA-1273 (triangular data points, lower panels). The cDNA synthesized by the two dilution approaches was amplified by PCR run in duplicate on the same plate with the identical experimental condition, but analyzed separately to obtain the Ct value in correspondence to each spike mRNA concentration. In both approaches, the outliers (blue or green data points in dot plots) were masked to obtain the best fitting lines with R^2 of >0.99. The best fitting standard lines (X= Ct and Y= Ln(C_{mRNA})) were almost in parallel with nearly identical slopes (red data points, right panel). Their distinction lay in the vertical intercepts, which were mainly ascribed to a discrepancy in DNA amplicon amount corresponding to the Ct value preset by the software at data analyses. Apparently, the former (circular data points) had a better sensitivity in the quantifying spike mRNA of the unknowns than the latter (triangular data points), which showed the clear deviation of the final three mRNA dilutions (green triangular data points) away from the fitting line. In a given experimental condition, the reverse transcription yields of cDNA are in proportion to target mRNA template amounts.[S3] Once mRNA was serially diluted past a certain threshold, the rarity of target mRNA available in reverse transcription reactions significantly lowered the reverse transcription efficiency.[S2] It gave rise to cDNA of low abundance, which in turn heightened the Monte Carlo effect on PCR amplification due to the low probability of primer annealing to target cDNA.[S1] Under these circumstances, the amplified products failed to truly reflect the cDNA abundance, leading to the outlier Ct values in correspondence to the final 3 dilutions of mRNA-1273. **(C)** Absolute spike mRNA quantification in various tissues was conducted by RT-PCR using the standard curve method. The unknowns were compared to the standard curve so as to extrapolate a value. The standard curve was constructed by 3-fold serial dilutions of cDNA reversely-transcribed from 10^4 pg/μL mRNA-1273 with corresponding spike mRNA of 10^4 - 0.0012 pg/μL (left panel). On a scatter plot (middle panel), there was a linear relationship between natural logarithm of spike mRNA template amount (C_{mRNA}) and cycle threshold (Ct). The equation of the best fitting line was: $Ln(C_{mRNA}) = -0.6789\ Ct + 15.608$ (right panel) to the exclusion of two left upper data points (blue, outliers, middle panel) that did not fit the trend line very well. This dataset of a standard trend line was obtained from a representative experiment of RT- PCR to quantify spike mRNA in trunk soft tissues of the fetuses with maternal 4.0 μg mRNA-1273 vaccination. Ln(C_{mRNA}) and their corresponding Ct values satisfied a linear relationship within the range of 0.0021 and 1111.1111 pg/μL mRNA-1273. Within this demarcated mRNA range (corresponding to Ct values between 32.0168 and 12.5616), samples' mRNA levels could be accurately determined via the linear equation by inputting the independent variables of Ct values.[S4] When a Ct value was equal to or higher than 33.1922 of negative controls (fetal soft tissues with gestational maternal saline injection), the sample was negative for spike mRNA. If a Ct value ranged between 32.0168 (corresponding to 0.0021 pg/μL spike mRNA) and 33.1922 (negative controls), the sample's mRNA level was recorded as <0.0021 pg/μL. The slope of −0.6789 in this equation was close to the theoretical slope of −0.6931.

Supplemental Reference:

S1. Bustin, S.A., and Nolan, T. (2004). Pitfalls of quantitative real-time reverse-transcription polymerase chain reaction. J Biomol Tech *15*, 155-166.

S2. Curry, J., McHale, C., and Smith, M.T. (2002). Low efficiency of the Moloney murine leukemia virus reverse transcriptase during reverse transcription of rare t(8;21) fusion gene transcripts. Biotechniques *32*, 768-775.

S3. Stahlberg, A., Kubista, M., and Pfaffl, M. (2004). Comparison of reverse transcriptases in gene expression analysis. Clin Chem *50*, 1678-1680.

S4. Nolan, T., Hands, R.E., and Bustin, S.A. (2006). Quantification of mRNA using real-time RT-PCR. Nat Protoc *1*, 1559-1582.

Supplemental tables were shown in Excel format as follows:

Table S1: Spike mRNA levels in offspring's blood, placenta, liver, trunk soft tissue and spleen after maternal vaccination with 4.0 μg mRNA-1273

Table S2: Spike mRNA levels in offspring's blood, placenta, liver, trunk soft tissue and spleen after maternal vaccination with 0.2 μg mRNA-1273

Table S3: Optic density of serum anti-spike IgG_{2a} allotypes detected by anti-mouse Igh-1a/b, anti-mouse Igh-1a and anti-mouse Igh-1b after gestationally maternal vaccination of 4 μg mRNA-1273

Table S4: Optic density of serum anti-spike IgG_{2a} allotypes detected by anti-mouse Igh-1a/b, anti-mouse Igh-1a and anti-mouse Igh-1b by 4 weeks postpartum after gestationally maternal vaccination of 0.2 μg mRNA-1273
