# Supplementary material for: mRNA-1273 is placenta-permeable and immunogenic in the fetus
Source: Mol Ther Nucleic Acids. 2025 Feb 17;36(1):102489. doi: 10.1016/j.omtn.2025.102489 (PMC11919431; doi:10.1016/j.omtn.2025.102489)
Supplement: Document S2. Article plus supplemental information [file mmc3.pdf]

Molecular Therapy
Nucleic Acids
Original Article

# mRNA-1273 is placenta-permeable and immunogenic in the fetus

Jeng-Chang Chen,[1,2] Mei-Hua Hsu,[3,4] Rei-Lin Kuo,[5,6,7] Li-Ting Wang,[6] Ming-Ling Kuo,[7,8] Li-Yun Tseng,[9] Hsueh-Ling Chang,[9] and Cheng-Hsun Chiu[3,4]

[1]School of Medicine, College of Medicine, Chang Gung University, Taoyuan 333, Taiwan; [2]Division of Pediatric Surgery, Department of Surgery, Chang Gung Memorial Hospital, Taoyuan 333, Taiwan; [3]Division of Pediatric Infectious Diseases, Department of Pediatrics, Chang Gung Memorial Hospital, Taoyuan 333, Taiwan; [4]Molecular Infectious Disease Research Center, Chang Gung Memorial Hospital, Taoyuan 333, Taiwan; [5]Department of Medical Biotechnology and Laboratory Science, College of Medicine, Chang Gung University, Taoyuan 333, Taiwan; [6]Research Center for Emerging Viral Infections, College of Medicine, Chang Gung University, Taoyuan 333, Taiwan; [7]Division of Allergy, Asthma, and Rheumatology, Department of Pediatrics, Chang Gung Memorial Hospital, Taoyuan 333, Taiwan; [8]Department of Microbiology and Immunology, Graduate Institute of Biomedical Sciences, College of Medicine, Chang Gung University, Taoyuan 333, Taiwan; [9]Pediatric Research Center, Chang Gung Memorial Hospital, Taoyuan 333, Taiwan

**COVID-19 mRNA vaccines are generally recognized as safe for gestational administration. However, their transplacental pharmacokinetics remain obscure. In this study, mRNA-1273 intramuscularly given to pregnant mice rapidly circulated in maternal blood and crossed the placenta within 1 h to spread in the fetal circulation. Although spike mRNA in fetal circulation faded away within 4–6 h, it could accumulate in fetal tissues, mainly the liver and get translated into spike protein. Transplacental mRNA-1273 proved immunogenic in the fetuses, as postnatally equipped with anti-spike immunoglobulin (Ig)M, paternal allotypic anti-spike $IgG_{2a}$, and heightened anti-spike cellular immunity. Gestationally administered, mRNA-1273 had a dose-dependent effect on its transplacental transfer and immunogenicity in the fetuses, with higher mRNA-1273 doses leading to increased transplacental mRNA-1273 passage and greater serum titers of endogenous anti-spike IgM/IgG generated by the fetuses. Thus, gestationally maternal mRNA-1273 vaccination might endow the newborns with not only passive but also active anti-spike immunity. Our results pose new insights into transplacental capacity of mRNA vaccines and their immunogenic potential in the fetuses, advancing our knowledge of mRNA medicine to protect the unborns against pathogens in perinatal life and broaden our horizons of prenatal mRNA molecular therapy.**

## INTRODUCTION

The two next-generation vaccines of Moderna mRNA-1273[1] and BioNTech BNT162b2,[2] based on SARS-CoV-2 spike protein-encoding mRNA strands packaged in lipid nanoparticles (LNPs),[3] have been widely used during and after the COVID-19 pandemic. They conferred over 90% efficacy against COVID-19 with a favorable safety profile in adults.[1,2] However, heightened pharmacovigilance pertaining to potential or unexpected embryotoxic/fetotoxic effects of brand-new medical products administered during pregnancy precluded gravid women from mRNA-LNP vaccination at the outset[4] even though COVID-19 during pregnancy tended to pose a higher risk for maternal or neonatal complications.[5] Since accumulated clinical data and observations supported the safety of mRNA vaccines for the mother and fetus,[5] mRNA-LNP vaccination prior to[6] or during pregnancy[7] has been highly recommended. However, the pharmacokinetics of mRNA-LNPs in gravid females remains shrouded in clouds, especially as to their transplacental capacity. Although LNPs were reported to enable *in vivo* vascular endothelial growth factor mRNA delivery to the placenta accompanied by its vasodilation,[8] neither vaccine mRNA nor mRNA-decoded spike protein could be detected in the placenta[9] and cord blood[10] sampled 2 days at least and mostly over weeks or even months after final maternal BNT-162b2 or mRNA-1273 vaccination. It brought to the notion that the placenta acted as the natural barrier to mRNA-LNPs, providing additional reassurance about the safety of mRNA vaccines during pregnancy. However, it was reported that mRNA-LNPs were swiftly cleared from the circulation during the first 24 h with the time required for 50% decrement of mRNA-LNP concentration ($T^1/_2$) in a range of 2.7–3.8 h,[11] implicating that transplacental mRNA-LNP transfer, if any, would most likely occur within 24 h after maternal vaccination. Moreover, mRNA-LNPs administered intravenously in fetal[12] or adult animals[13] underwent rapid systemic spread with preferential LNP accumulation and peak mRNA functionality in the liver within 4 h followed by decreasing protein levels at 24 h after injection or translation ceasing on day 2. Taken together, it seemed premature to negate transplacental mRNA-LNP transfer on the basis of undetectable vaccine mRNA or its products in belatedly collected fetal blood or placenta that was even not favorable to harboring mRNA-LNPs or spike proteins. We conducted this murine study

Received 6 September 2024; accepted 13 February 2025;
https://doi.org/10.1016/j.omtn.2025.102489.

**Correspondence:** Cheng-Hsun Chiu, Department of Pediatrics, Chang Gung Memorial Hospital, No. 5, Fu-Shin Street, Kweishan, Taoyuan 333, Taiwan.
**E-mail:** chchiu@adm.cgmh.org.tw


© 2025 The Author(s).
Published by Elsevier Inc. on behalf of The American Society of Gene and Cell Therapy.
This is an open access article under the CC BY license (http://creativecommons.org/licenses/by/4.0/).

to reappraise the transplacental capacity of mRNA-1273 and scrutinize its immunogenicity in the fetuses.

## RESULTS

### Detection of transplacental polyethylene glycol lipid

Following a single-dose intramuscular injection of 4 μg mRNA-1273 into gestational day 14 (GD14) pregnant mice, the fetuses were delivered and euthanized at selected time points to search fetal blood for transplacental LNPs, using anti-polyethylene glycol (PEG) antibodies. PEGylated LNPs swiftly moved into the maternal bloodstream and efficiently crossed the placenta to spread in fetal circulation within 30 min (Figure 1A). However, they faded away in maternal circulation within 3–24 h but lasted over time in the fetal circulation for at least 7 days, indicating slower PEG breakdown in the fetuses than the dams. Further enzyme-linked immunosorbent assay (ELISA) confirmed that PEG levels stayed steady in fetal sera within 3 h after maternal 4 μg mRNA-1273 vaccination, and dropped significantly at 6-h and 1- to 3-day time points (Figure 1B). PEG in the offspring's sera was barely found on days 7–11 post-maternal vaccination, and no longer detectable by days 14–18. There was no detectable PEG in fetal placenta, liver, and soft tissues by ELISA at any of the time points examined (data not shown). Notably, a reduction of maternal mRNA-1273 doses caused a decline in serum PEG levels in the fetuses within 3 h after maternal vaccination (Figure 1C).

### Detection of transplacental spike mRNA

We then examined whether transplacental LNP transfer was coupled with vaccine active substance of SARS-CoV-2 spike mRNA by reverse transcription PCR (RT-PCR) (Figure S1). After maternal intramuscular vaccination of 4 μg mRNA-1273, spike mRNA entered the maternal circulation and crossed the placenta to fetal blood within 1 h, whereas transplacental spike mRNA shortly became undetectable in fetal circulation by 4–6 h (Table S1). Thus, spike mRNA was more liable to degradation than PEG in fetal blood. Transplacental spike mRNA mainly accumulated in fetal livers, and also dwelt in fetal placentas and trunk soft tissues (Figure 1D; Table S1). Spike mRNA might persist in offspring's liver and spleen at least until postnatal 3 weeks (Table S1). Notably, immunofluorescence staining demonstrated the lodging of PEGylated LNPs and spike protein in fetal liver cells (Figure 1E). Taken together, transplacental mRNA-1273 transfer came along with mRNA-decoded protein expression in the fetus. In dams vaccinated with 0.2 μg mRNA-1273, vaccine mRNA also rapidly spread to the maternal circulation, accrued to the placenta, and distributed to the fetus (Table S2). Although levels of spike mRNA in fetal placentas did not differ between 0.2 and 4.0 μg mRNA-1273 administered to the dams, low-dose mRNA-1273 gave rise to less spike mRNA accumulation in fetal livers than high-dose ones (Figure 1F). Overall, transplacental mRNA-1273 transfer exhibited a maternally dose-dependent response, with higher maternal mRNA-1273 doses resulting in greater levels of PEG in the circulation and spike mRNA in the liver of the fetus. However, comparable levels of spike mRNA in fetal placentas between high- and low-dose mRNA-1273 given to the dams might suggest placental trapping of mRNA-1273 before reaching the fetus.

### Examination of anti-spike $IgG_1$/$IgG_{2a}$ with their virus-blocking efficacy

To elucidate the immunological consequences of transplacental mRNA-1273 transfer in the fetus, we scrutinize the influence of mRNA-1273 doses given intramuscularly to pregnant mice on serum anti-spike immunoglobulin levels of dams and pups. After maternal mRNA-1273 vaccination at the same dose of 0.2, 1.0, 2.0, or 4.0 μg on GD14 and GD17, the dams and their pups were examined for serum anti-spike immunoglobulin (Ig) levels 1 month after delivery. The vaccinated mothers significantly generated anti-spike $IgG_1$/$IgG_{2a}$ in the absence of a dose-responsive fashion, ranging respectively around 100–200 ng/mL and 20–40 μg/mL with relatively steady levels over a postnatal period of 3 months (Figure 2A). However, mRNA-1273 given to pregnant mice exerted a dose-dependent effect on offspring's serum anti-spike $IgG_1$/$IgG_{2a}$ levels, which showed a dwindling trend over time (Figure 2B). Virus-blocking efficacy of maternal sera was as high as 1024- to 2048-fold dilutions at least within postnatal 2–3.5 months, whereas the pup's sera at 2 months old had lower neutralization activity, which even vanished by 3.5 months old (Figure 2C). It was essentially consistent with the distinct durability of serum anti-spike IgG between the dams (Figure 2A) and their offspring (Figure 2B). The discordance between the first month anti-spike $IgG_1$/$IgG_{2a}$ levels of mothers and their offspring in response to mRNA-1273 doses used to vaccinate the dams might have relevance to transplacental mRNA-1273 transfer in a maternally dose-dependent manner (Figures 1C and 1F; Tables S1 and S2). It called into question whether transplacental mRNA-1273 transfer was not only maternally dose-dependent but also exerted a dose-dependent effect on triggering the fetal immune system to generate endogenous anti-spike IgG.

### Analyses of anti-spike $IgG_{2a}$ allotypes in offspring

Investigations proceeded to assess the derivation of the offspring's anti-spike $IgG_{2a}$ after maternal mRNA-1273 vaccination, using two allelic forms (Igh-1a and Igh-1b) of the Igh-1 ($IgG_{2a}$, γ2a constant region). The mouse BALB/c strain possesses the $IgG_{2a}$ of Igh-1a haplotype, whereas the C57BL/6 strain belongs to the Igh-1b haplotype. C57BL/6 females were mated to BALB/c males, and then given 4 μg mRNA-1273 vaccination respectively on GD14 and GD17. Postnatally, the offspring (BALB/c male × C57BL/6 female, F1) co-expressed both Igh-1a and Igh-1b haplotypes of anti-spike $IgG_{2a}$ at the age of 4 weeks despite fading-out of the Igh-1a haplotype by 8 weeks old (Figures 3A and 3B; Table S3). It provided the direct molecular evidence that the offspring were equipped with endogenous anti-spike $IgG_{2a}$, which could not originate from anything other than the offspring's B cell clones selectively expressing paternal Igh-1 allotype. Clearly, the offspring born to dams with gestational mRNA-1273 vaccination had been immunized by spike protein and additionally armed with endogenous anti-spike IgG. Incidentally, serum anti-spike $IgG_{2a}$ of all C57BL/6 dams, mated to BALB/c males, was found to contain Igh-1a haplotype of fetal origin (Table S3), indicating a reverse direction of fetal-to-maternal anti-spike $IgG_{2a}$ transfer. Thus, transplacental IgG transfer could be bidirectional. When gestational C57BL/6 dams were vaccinated with 0.2 μg

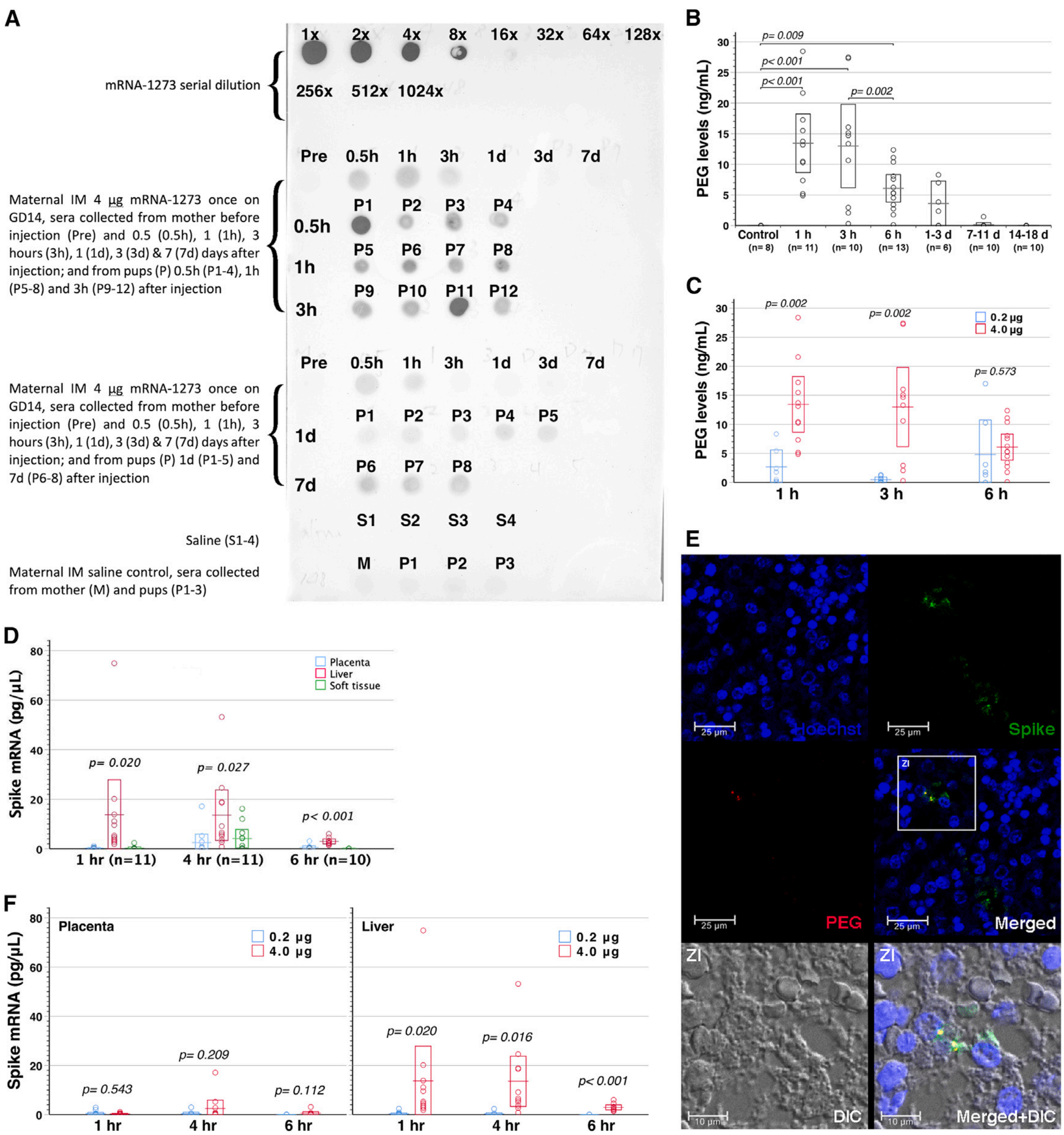


**Figure 1. Transplacental mRNA-1273 transfer after maternal mRNA-1273 vaccination during pregnancy**

(A) GD14 FVB/N mothers, intramuscularly (IM) vaccinated with a single-dose mRNA-1273 of 4.0 μg, were subjected to serum collection before vaccination (Pre), and at indicated time points of 0.5–3 h and 1–7 days after injection. Their pups were delivered for serum sampling at the same time points. Immunodot blot assay demonstrated transplacental PEGylated LNP transfer. (B) ELISA disclosed that fetal sera contained significantly higher PEG levels at the time points of 1 h, 3 h, and 6 h after maternal mRNA-1273 vaccination than those with maternal saline injection (control, ANOVA with least significant difference (LSD) multiple comparison). A significant decrease of serum PEG levels occurred between 3 h and 6 h. Although PEG remained measurable in certain pups of groups 1–3 d and 7–11 d, their mean levels did not differ from that of saline controls. On days 14–18, PEG was completely absent in all neonatal sera, identical to saline controls. (C) At the time points of 1 h and 3 h following maternal vaccination, 4.0 μg mRNA-1273 led to higher PEG levels in fetal sera than a dose of 0.2 μg. (D) Spike mRNA in fetal placenta, liver, and soft tissue was quantified by RT-PCR after maternal 4 μg mRNA-1273 vaccination (dams 234, 235, and 236 in Table S1). Spike mRNA levels of "(−)" and "< 0.021" were input as "0" and "0.021," respectively in building this chart. Spike mRNA significantly dominated in fetal liver of groups 1, 4, and 6 h (ANOVA with LSD multiple comparison). (E) Immunostaining disclosed intracellular PEGylated LNPs and spike protein in fetal liver 6 h after maternal 4.0 μg mRNA-1273 vaccination. DIC: differential interference contrast. ZI: zoom-in. (F) At the time points of 1, 4, and 6 h after maternal mRNA-1273 vaccination, levels of spike mRNA in fetal placentas did not differ between 4.0 and 0.2 μg mRNA-1273 used to vaccinate the dams (Tables S1 and S2), whereas 4.0 μg mRNA-1273 led to significantly greater spike mRNA accumulation in fetal livers than 0.2 μg mRNA-1273. Error bar charts display the boxed areas of 95% confidence intervals for the means as box-crossing horizontal lines.

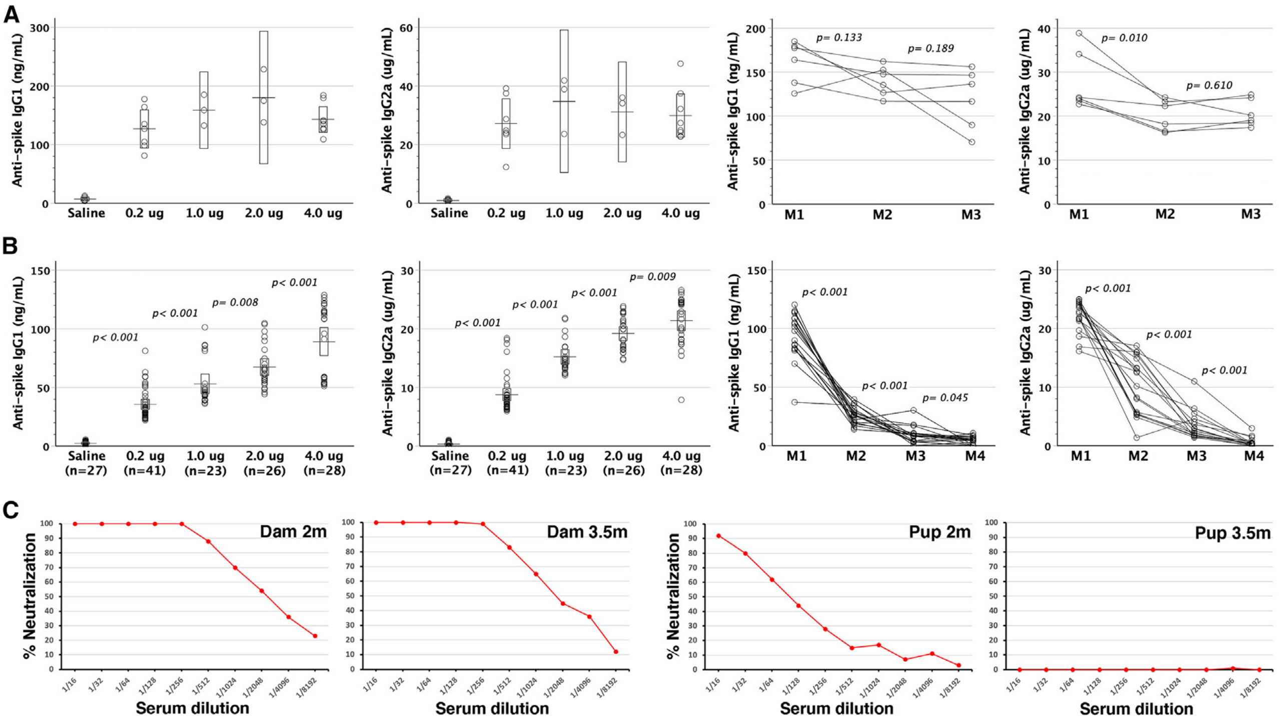


**Figure 2. Anti-spike $IgG_1$/$IgG_{2a}$ with virus-blocking efficacy in dams and their offspring after gestational mRNA-1273 administration**

Pregnant FVB/N mice were intramuscularly vaccinated by the same doses of mRNA-1273 (0.2, 1, 2, or 4 μg) on GD14 and GD17. (A) One month after delivery (M1), all four mRNA-1273 doses elicited significant levels of serum anti-spike $IgG_1$/$IgG_{2a}$ in dams and (B) pups ($p < 0.001$, ANOVA), as compared with their saline controls. There were dose-responsive anti-spike $IgG_1$/$IgG_{2a}$ levels in pups rather than dams (multiple comparisons by Fisher's LSD post hoc test). The dams ($n = 6$) kept steady anti-spike $IgG_1$/$IgG_{2a}$ titers in sera within postnatal 3 months (M1–M3) except for an initial drop of anti-spike $IgG_{2a}$ levels ($p = 0.010$, pairwise comparison) at M2, whereas anti-spike $IgG_1$/$IgG_{2a}$ in pups' sera ($n = 18$) gradually faded away by M3–M4. The interconnected circles at different time points were the data of $IgG_1$/$IgG_{2a}$ levels collected from an individual mouse. (C) Virus-blocking efficacy of maternal and offspring sera was evaluated by pseudovirus neutralization assays and shown in a representative mother and its offspring. Postnatal 2- and 3.5-month maternal sera had the neutralization titers of 2048- and 1024-fold dilutions, respectively, whereas neutralization activity of offspring sera was 64-fold at 2 months old but vanished by 3.5 months old. Error bar charts display the boxed areas of 95% confidence intervals for the means as box-crossing horizontal lines.

mRNA-1273 twice, their offspring barely generated anti-spike $IgG_{2a}$ of Igh-1a haplotype despite high titers of Igh-1b allotypic anti-spike $IgG_{2a}$ in sera (Figure 3C; Table S4). These results pointed to a dose-responsive relationship between mRNA-1273 doses used to vaccinate the dams and serum titers of endogenous anti-spike IgG in the fetuses. As a consequence, mRNA-1273 had a maternally dose-dependent effect on not only its transplacental capacity but also its immunogenicity as to the productivity of endogenous anti-spike IgG in the fetuses.

## Assessment of anti-spike IgM and cellular immunity

Being placenta-impermeable, anti-spike IgM in offspring was measured to reconfirm fetal immunization by transplacental mRNA-1273. Maternal vaccination with either 0.2 or 4.0 μg mRNA-1273 gave rise to heightened anti-spike IgM levels in offspring by the age of 4 weeks (Figure 3D). High-dose mRNA-1273 led to higher serum titers of fetal anti-spike IgM than low-dose ones. Vaccinated dams and their offspring were further examined for cellular immunity to spike protein by the readout of incorporated tritium into lymphocytes. Both compared favorably in spike protein-specific lymphocyte proliferation (Figures 4A and 4B) with their respective saline control counterparts. Additionally, spike-reactive interferon (IFN)-γ- and interleukin (IL)-2-secreting T cells were enumerated by enzyme-linked immunospot assay (ELISpot), proving at significantly heightened frequencies as opposed to their saline controls (Figures 4C and 4D). Altogether, maternal mRNA-1273 vaccination during pregnancy might trigger adaptive immunity against spike protein in dams and their pups.

## Immunological outcome of direct fetal exposure to mRNA-1273

To further validate the immunogenic effects of mRNA-1273 on preimmune fetuses, we directly subjected GD14 murine fetuses to intraperitoneal mRNA-1273 injection. Postnatally, fetal recipients exhibited heightened titers of serum anti-spike $IgG_1$/$IgG_{2a}$ (Figures 5A and 5B), which decreased gradually within the postnatal 3 months. Their lymphocytes also proliferated specifically in response to SARS-CoV-2 spike protein (Figure 5C) in association with a heightened frequency of spike-reactive IFN-γ and IL-2-secreting T cells (Figure 5D). These results signified fetal immunoreactivity to

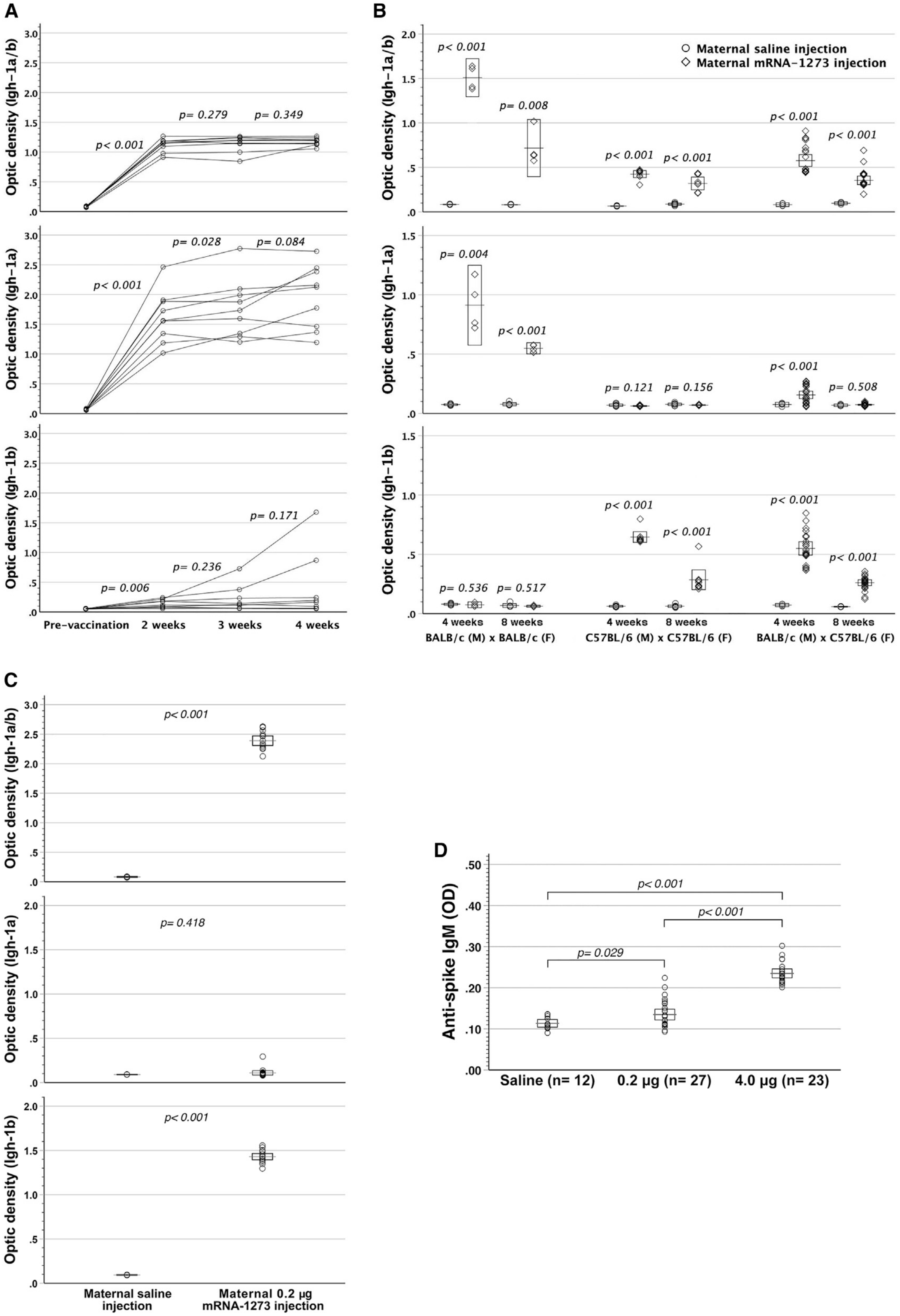


*(legend on next page)*

mRNA-1273 administered *in utero* even before full T cell development.

## DISCUSSION

Maternal mRNA COVID-19 vaccination during pregnancy offered a "two-for-one" deal to protect mothers as well as their infants.[7] This added infant protection has long since been attributed to transplacental transfer of vaccine-elicited maternal anti-spike antibodies,[4,7] likened to maternal vaccination against influenza, tetanus, diphtheria, and pertussis.[14] In this study, murine placentas proved permeable to mRNA-1273 along with mRNA-decoded spike protein translation in GD14 murine fetuses, which lacked functionally competent T cells as murine T cell receptors were not expressed until GD17.[15] However, vertical mRNA-1273 transmission made maternal mRNA-1273 vaccination immunogenic rather than tolerogenic in developing fetuses. It is in line with an immunization event arising after artificial fetal exposure to foreign peptides,[16,17] wherein fetal macrophages sequestered endocytosed antigens and differentiated toward dendritic cells to instruct well-developed T cells later on in life.[16] Thus, the developing immune system of fetuses born to mothers with gestational mRNA-1273 vaccination still had an active role in protecting against pathogens.

Mammalian placentas are categorized into epitheliochorial, endotheliochorial, and hemochorial types on the basis of the cell layers intervening between maternal and fetal circulations,[18] known as interhemal barriers that influence placental permeability of animal species. Both humans and mice possess hemochorial placentas with similar cell types of placental trophoblasts[19,20] to mediate materno-fetal exchange. Hemochorial placentation lacks interhemal barriers of uterine endometrium (including its epithelia, stroma, and vascular endothelia), leading to direct immersion of fetal trophoblast layers in maternal blood. It benefits bio-substance exchange between mothers and their fetuses by providing direct access of fetal trophoblasts to maternal blood.[21] In light of the identical interhemal barriers in the placenta, the mouse model should be appropriate for simulating placental exchange of bio-substances in humans. However, one should exercise caution in extrapolating the findings of transplacental mRNA-1273 transmission from this murine study to human subjects since there is a distinction in histoarchitectures between murine and human placentas, evidenced by three-layered trophoblasts (hemotrichorial) at the murine materno-fetal interface in contrast to one-layered syncytiotrophoblast (hemomonochorial) of human placenta.[20] It remained unclear whether the two extra trophoblast layers in murine placentas exerted negative or positive impacts on materno-fetal exchange of bio-substances, let alone mRNA-1273. This murine study demonstrated that mRNA-1273 possessed the dose-dependent transplacental capacity in gravid dams and exhibited immunogenic potential in the fetuses. These findings urge the need to reappraise transplacental capacity of mRNA-LNPs and clarify the status of immunoreactivity to spike protein in human fetuses or infants with gestational maternal mRNA COVID-19 vaccination. The information obtained will have a profound influence on the COVID-19 vaccination strategy in infants born to gestationally vaccinated mothers.

mRNA vaccines exhibited good safety profiles in humans[1,2] and even pregnant individuals.[22–24] However, the positive safety outcomes in the clinical arena could not alleviate the apprehensions about the potential genotoxicity such as genome integration, oncogenesis, or germline transmission,[25–27] which was fueled by enduring biodistribution of vaccine mRNA[28,29] or its product[30,31] in post-marketing studies. These inconvenient findings somewhat reflected the rapid marketing authorization of mRNA vaccines with incomplete preclinical studies due to the urgent health needs in the face of a public health crisis caused by the COVID-19 pandemic.[25] This murine study filled the void in transplacental pharmacokinetics of mRNA vaccines, which has been missing in preclinical studies even though mRNA vaccines themselves involved several new biotechnologies. In this research, mRNA-1273 did not pose discernible safety issues in pregnant mice and their pups. However, the proof of transplacental mRNA-1273 transmission with enduring mRNA retention in the offspring's liver or spleen inevitably aroused an interest in the genotoxic effects of mRNA vaccines on the developing fetus, where heightened activities of cell multiplication and specialization potentially created genomic instability[32,33] to render the fetus vulnerable to the integration of exogenous genetic elements.[34,35] Considering the occurrence of SARS-CoV-2 RNA retro-integration into the human cell genome,[26] the risk of long-term genotoxicity in the offspring born to mRNA-vaccinated mothers cannot be overlooked.

Given the success of mRNA COVID-19 vaccines and today's biotech landscape, there is a prospect of extending mRNA-LNP technology to the genetic diseases with defective/missing proteins or enzymes such as cystic fibrosis, propionic acidemia, and

**Figure 3. Analyses of anti-spike $IgG_{2a}$ allotypes and anti-spike IgM in offspring born to the dams with gestational mRNA-1273 vaccination**
(A) After mRNA-1273 vaccination, BALB/c (Igh-1a) × C57BL/6 (Igh-1b) F1 mice ($n$ = 9) significantly secreted anti-spike $IgG_{2a}$ (Igh-1a/b) in sera within 2–4 weeks (pairwise comparison). Igh-1a haplotype dominated the allotypes of anti-spike $IgG_{2a}$. (B) C57BL/6 females (F) mated to BALB/c males (M) were vaccinated with 4 μg mRNA-1273 twice on GD14 and GD17. Both paternal Igh-1a and maternal Igh-1b allotypic anti-spike $IgG_{2a}$ significantly showed up in BALB/c (M) × C57BL/6 (F) F1 mice at 4 weeks old ($p < 0.001$) despite undetectable paternal Igh-1a allotype by 8 weeks old ($p = 0.508$). (C) In the case of 0.2 μg mRNA-1273 vaccination in C57BL/6 pregnant mice, BALB/c × C57BL/6 F1 offspring ($n$ = 17) did not compare favorably in serum anti-spike $IgG_{2a}$ of Igh-1a ($p = 0.418$) with their saline controls ($n$ = 5) but owned significantly higher levels of Igh-1b ($p < 0.001$) allotype than the controls by their age of 4 weeks. (D) After maternal vaccination with either 0.2 or 4.0 μg mRNA-1273 twice, offspring showed significantly heightened levels of serum anti-spike IgM by their age of 4 weeks, as compared with the controls with maternal saline injection. Besides, 4.0 μg mRNA-1273 given to the dams elicited higher serum titers of anti-spike IgM in offspring than 0.2 μg mRNA-1273 ($p < 0.001$). OD: optic density at 450 nm. Error bar charts display the boxed areas of 95% confidence intervals for the means as box-crossing horizontal lines.

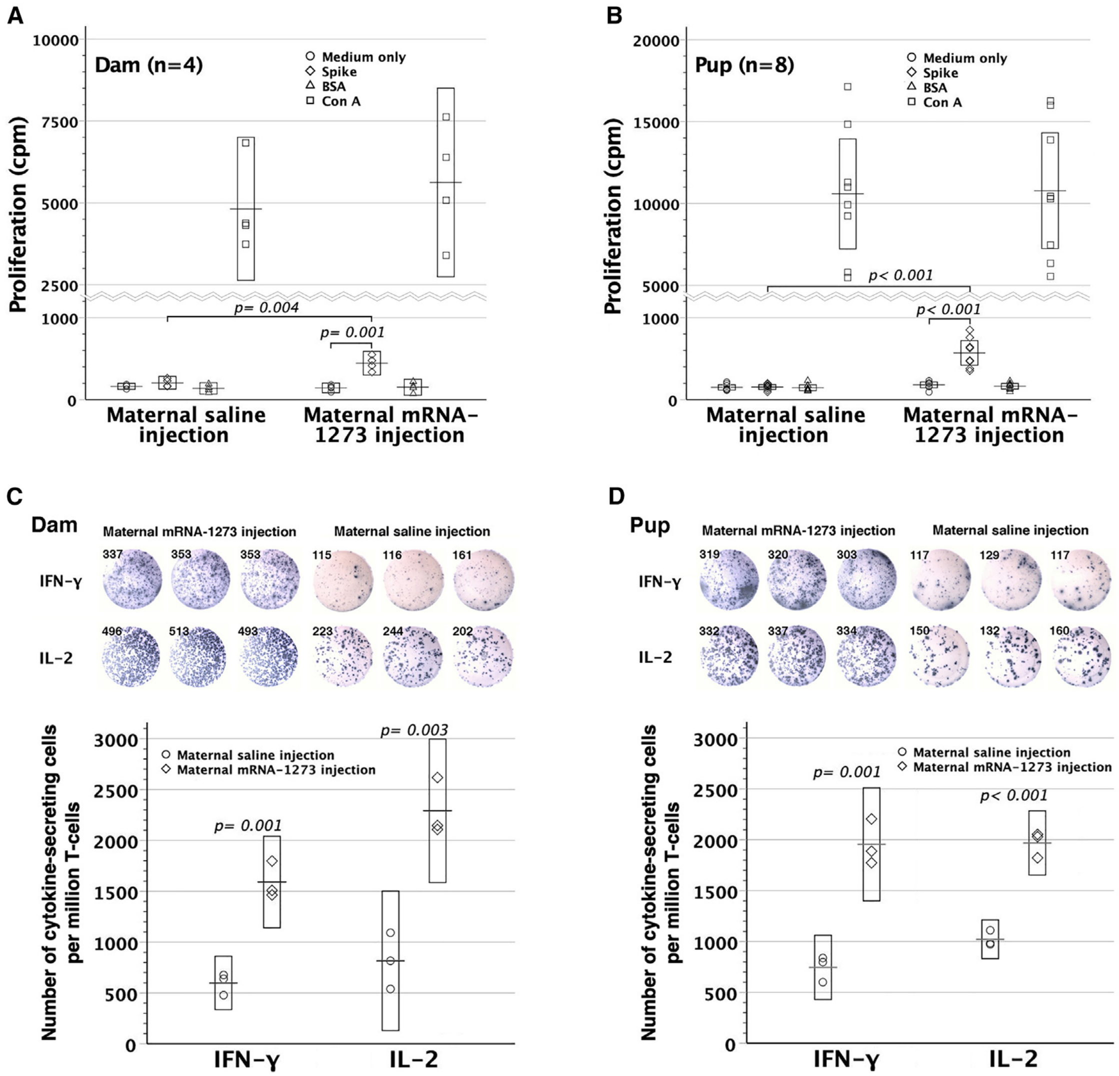


**Figure 4. Anti-spike cellular immunity in dams and their pups after maternal mRNA-1273 vaccination during pregnancy**

(A and B) After maternal vaccination with 4 μg mRNA-1273 twice, the dams and pups were examined for spike-specific lymphocyte proliferation by the readout of incorporated tritium *in vitro*. Splenic lymphocytes of both dams ($p = 0.001$) and pups ($p < 0.001$) proliferated specifically in response to spike, as opposed to those with maternal saline injection. Besides, the dams ($p = 0.004$) and pups ($p < 0.001$) with maternal mRNA-1273 vaccination were superior in spike-specific lymphocyte proliferation to their respective saline controls. Bovine serum albumin (BSA) was the third-party stimulators, and Con-A was a mitogen to non-specifically stimulate T cells. (C and D) IFN-γ- and IL-2 ELISpot images in triplicate shown were from a representative dam and pup with maternal mRNA-1273 (4 μg) or saline vaccination during pregnancy. Both groups exhibited heightened frequencies of IFN-γ- and IL-2-secreting T cells, as compared with their respective control counterparts. Error bar charts display the boxed areas of 95% confidence intervals for the means as box-crossing horizontal lines.

phenylketonuria.[3] These candidate diseases if diagnosed prenatally can be managed by prenatal mRNA therapies before the onset or in the early stage of irreversible pathology to minimize disease morbidity and mortality, and achieve high therapeutic efficacy. In consideration of transplacental mRNA-1273 passage, the unmet need of fetal mRNA therapies may be fulfilled simply through maternal mRNA-LNP administration in case of no harm to the mothers, but the potential immunogenicity of mRNA-decoded peptides must be taken into consideration even in pre-immune fetuses. However, it is better to note that the ability of LNPs to deliver mRNA and accumulate within desired tissues or organs varied with changes in LNP chemistry.[12,36] Thus, modifications in lipid excipients used for mRNA-LNP formulations might affect their transplacental capacity and warrant an evaluation of their transplacental properties.

In this era of mRNA medicine, the new insights into transplacental pharmacokinetics of mRNA vaccines and immunogenic potential

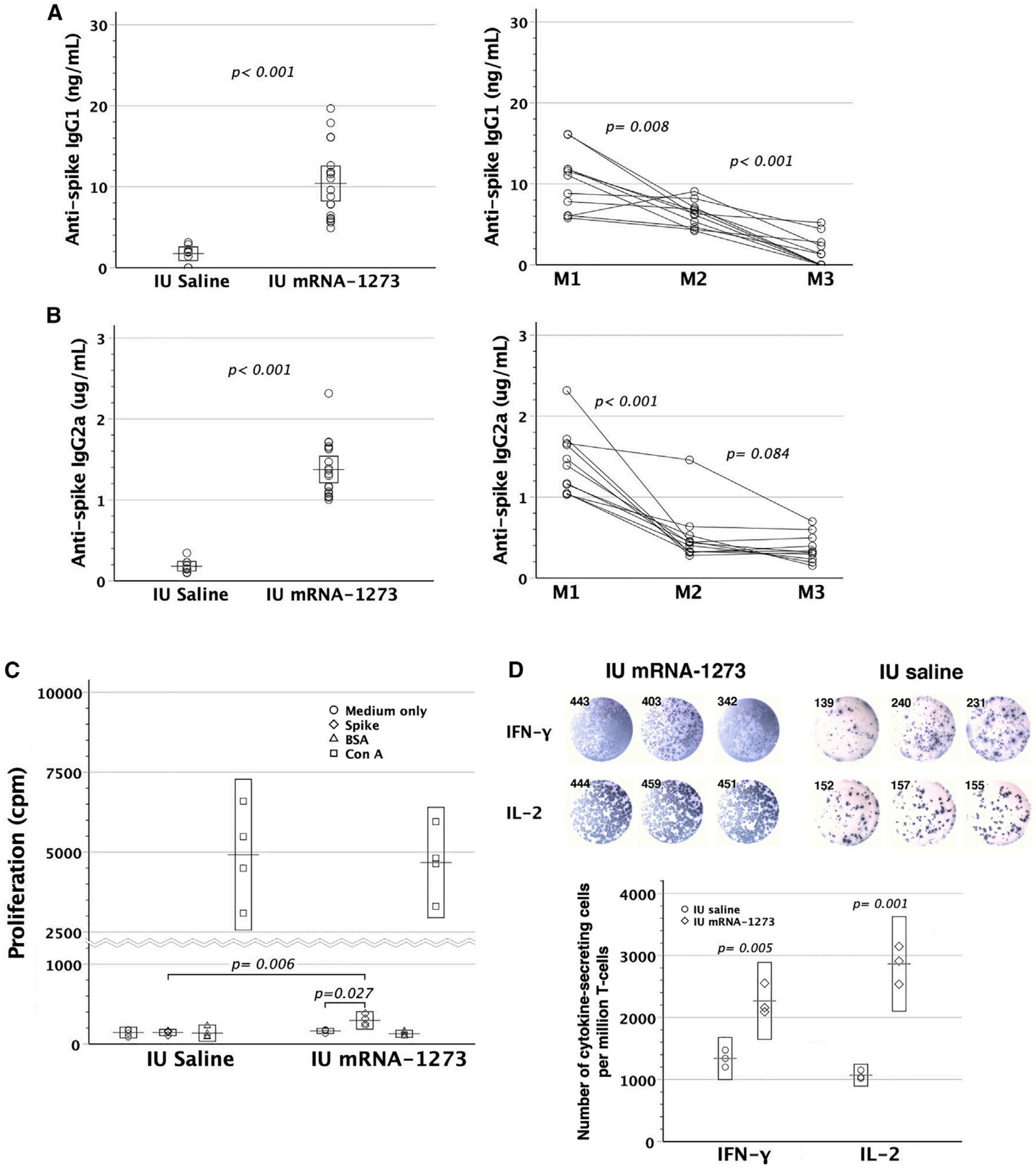


**Figure 5. Immunological consequences of *in utero* mRNA-1273 injection**

GD14 FVB/N fetuses were subjected to intraperitoneal injection of mRNA-1273 (IU mRNA-1273, $n = 19$). (A and B) Postnatally, serum anti-spike $IgG_1/IgG_{2a}$ was examined at the age of 1 month. IU mRNA-1273 led to significantly higher titers of anti-spike $IgG_1/IgG_{2a}$, as compared with *in utero* saline injection (IU saline, $n = 9$). Serum anti-spike $IgG_1/IgG_{2a}$ gradually decreased within postnatal 3 months. Circles interconnected by a line represent $IgG_1/IgG_{2a}$ levels measured at 1 (M1), 2 (M2), and 3 (M3) months old from an individual mouse ($n = 11$). (C) Lymphocyte proliferation in response to spike protein was measured by the readout of incorporated tritium ($n = 4$) as counts per minute (cpm). Medium only was used as background controls, BSA as third-party stimulators, and Con-A as a mitogen to stimulate the T cell population. IU mRNA-1273 significantly proliferated specifically in response to spike protein ($p < 0.027$), whereas IU saline ($n = 4$) failed to show lymphocyte proliferation under spike protein stimulation. There was a significant difference in lymphocyte proliferation under spike protein stimulation between IU mRNA-1273 and IU saline ($p < 0.006$). Rectangles within a dataset represent 95% confidence intervals for the means, which are shown as transverse lines crossing the rectangles. (D) Spike-reactive IFN-$\gamma$- and IL-2-secreting cells of splenic lymphocytes were enumerated by ELISpot. Figures showed the spots with their counts from the representative mice of IU mRNA-1273 and IU saline. The frequency of spike-reactive IFN-$\gamma$- and IL-2-secreting T cells was calculated by the mean of ELISpot readouts (triplicates) divided by the $CD3^+$ cell ratio of splenic lymphocytes in each individual mouse.

of mRNA-decoded protein in the fetuses may advance our knowledge to better protect the unborns against pathogens in perinatal life and broaden our horizons of prenatal mRNA therapeutics.

## MATERIALS AND METHODS

### Mice

Inbred FVB/N, BALB/c (Igh-1a of $IgG_{2a}$, γ2a constant region) and C57BL/6 (Igh-1b of $IgG_{2a}$, γ2a constant region) mice were purchased from National Laboratory Animal Center (Taipei, Taiwan) at the age of 6–8 weeks. Animals were housed in the Animal Care Facility at Chang Gung Memorial Hospital (CGMH) under the standard guidelines from "Guide for the Care and Use of Laboratory Animals" and with the approval of the CGMH Committee on Animal Research. Females were caged with males in the afternoon and checked for vaginal plugs the following morning. The day the plug was observed was designated as day 0 of the pregnancy.

### Harvest of fetal tissues

Under anesthesia for pregnant mice, midline laparotomy was performed to expose the uteri. The fetuses were delivered through hysterotomy and immediately washed with saline. After decapitation, fetal blood was collected by pipetmans. Then, fetal placenta, liver, and trunk soft tissues were obtained. Samples were stored in RNAlater solution at −80°C for downstream analyses or subjected to homogenization in organic solvents of ethanol or dimethyl sulfoxide (DMSO) for PEG extraction.

### mRNA-1273 vaccination in pregnant mice

Pregnant mice received intramuscular (thigh) injection of mRNA-1273 on their GD14 and GD17, each at the same dose of 0.2, 1, 2, or 4 μg, diluted in 100 μL saline. Postnatally, sera of the dams and their offspring were sampled periodically for downstream experimental analyses. For mRNA-1273 component tracking in the fetuses, a single dose of 0.2 or 4 μg mRNA-1273 was intramuscularly given to the mothers on their GD14. The fetuses were then delivered by cesarean at indicated time points after maternal vaccination to harvest fetal tissues and placentas for downstream analyses.

### *In utero* injection of mRNA-1273

Under anesthesia, the uteri of GD14 pregnant mice were exposed through a vertical laparotomy. A 60-μm glass micropipette with beveled tip was used to inject 0.05–0.1 μg of mRNA-1273 in 5 μL saline into the peritoneal cavities of all fetuses at a litter via *trans*-uterine approach. The control mice received *in utero* saline injection. Murine abdomen was closed in two layers by 5-0 silk suture. Then, mice were housed in an undisturbed room without bedding changes for 1 week. Pups were weaned at 3 weeks of age.

### Immunodot blot assay to detect LNPs of mRNA-1273

This method was modified from the fat blot assay by Munnik and Wierzchowiecka[37] to semiquantitatively detect mRNA-LNPs. Mouse sera and serially diluted mRNA-1273 (1 μL for each sample) were spotted onto a nitrocellulose membrane (0.45 NC, Amersham Protran). The membrane was first blocked with 5% milk in Tri-buffered saline containing 0.05% Tween 20 (TBST) for 1 h on a rotating shaker and then incubated with anti-polyethylene glycol (PEG) antibody (1:3,000, PEG-B-47, ab51257, Abcam, reacting only with conjugated forms) for 2 h. After washing with TBST three times, the membrane was treated with peroxidase-conjugated goat anti-rabbit IgG (1:5,000, AP132P, Sigma-Aldrich) for 2 h, followed by Immobilon Western Chemiluminescent HRP Substrate (Millipore) for 2 min. Finally, the blots were subjected to chemiluminescence imaging detection (UVP Chemstudio). Positive controls were 2-fold serial dilutions of mRNA-1273, and negative controls included saline and maternal/pups' sera collected after maternal saline injection.

### RT-PCR to quantify spike mRNA

RNA was isolated from tissue samples of fetuses with maternal mRNA-1273 vaccination using GeneJET RNA Purification Kit (Thermo Fisher Scientific) according to the manufacturer's protocol. RNA concentration was determined using Nanodrop. RNA samples of 500 ng were reversely transcribed into cDNA using PrimeScript RT reagent Kit (TaKaRa Bio). Primers used to detect target cDNA were as follows:[10,38] Forward primer: AACGCCACCAACGTGGTCATC. Reverse primer: GTTGTTGGCGCTGCTGTACAC. Bio-Rad iQ5 real-time PCR detection system and 2xSYBR qPCR Mix (BioTools) were used for PCR: 30 s at 95°C followed by 40 cycles of 5 s at 95°C and 20 s at 60°C. All samples (2 μL) were run in duplicate as 20-μL reactions. For setup of spike mRNA standard curves, cDNA reversely transcribed from 100 ng/μL mRNA-1273 was serially diluted in a 1:2 ratio. These 3-fold serial dilutions of 2-μL cDNA samples were further 10× diluted to 20 μL (corresponding to $10^4 - 0.0021$ pg/μL of spike mRNA) in PCR amplification. Negative results were determined by the Ct values of tissue samples from the fetuses with maternal saline injection.

### Determination of serum anti-spike $IgG_1$, $IgG_{2a}$, and IgM levels

ELISA microtiter plates (Corning, Corning, NY, USA) were first coated with 25 ng/mL and 50 ng/mL SARS-CoV-2 spike protein (GTX02774-pro, GeneTex) respectively for the measurement of mouse anti-spike $IgG_1$ and $IgG_{2a}$/IgM levels. The wells were blocked with 3% bovine serum albumin (BSA, Sigma-Aldrich) in PBS, and incubated with 100 μL of diluted samples. In each well, biotinylated anti-mouse $IgG_1$ (Clone RMG1-1, BioLegend, San Diego, CA, USA) was used for $IgG_1$ detection, biotinylated anti-mouse $IgG_{2a}$ (Clone RMG2a-62, BioLegend) for $IgG_{2a}$ detection, and biotinylated anti-mouse IgM (Clone RMM-1, BioLegend) for IgM detection. Subsequently, streptavidin-horseradish peroxidase (HRP, Sigma-Aldrich) was added to the wells. Then, the reaction was developed by adding 100 μL NeA-blue tetramethylbenzidine substrate (TMB) (Clinical Science Products, Mansfield, MA, USA) and stopped with 2M $H_2SO_4$. The optical density at 450 nm was read using an ELISA reader. Serum anti-spike $IgG_1$ and $IgG_{2a}$ levels were determined by the standard curves of mouse monoclonal anti-SARS-CoV-2 spike $IgG_1$ (1A9, GTX632604, GeneTex) and mouse monoclonal anti-SARS-CoV-1/2 S Protein $IgG_{2a}$ (clone 2B3E5, Sigma-Aldrich),

respectively. Serum anti-spike IgM titers were recorded as the values of optical density.

### Determination of anti-spike $IgG_{2a}$ allotypes

Igh-1a and Igh-1b allotypes of $IgG_{2a}$ were used to examine whether the pups (BALA/c [Igh-1a] male × C57BL/6 [Igh-1b] female, F1) generated endogenous anti-spike $IgG_{2a}$ after maternal intramuscular vaccination of 0.2 or 4.0 μg mRNA-1273, respectively, on GD14 and GD17 during pregnancy. ELISA was performed as described above, using 10× diluted serum samples treated with primary antibodies of biotinylated anti-mouse IgG2a (1:20,000, Clone RMG2a-62, reacting with both Igh-1a and Igh-1b [Igh-1a/b] haplotypes), biotinylated anti-mouse Igh-1a (1:2,000, clone 8.3, BD Pharmingen) or biotinylated anti-mouse Igh-1b (1:2,000, clone 5.7, BD Pharmingen), respectively. The color developed was read at optic density of 450 nm. Controls included BALB/c (male) × BALB/c (female) and C57BL/6 (male) × C57BL/6 (female) F1 mice.

### Quantification of PEG by sandwich ELISA

Sera or supernatants of tissue homogenates from murine fetuses were subjected to PEG quantification by ELISA kits (Life Diagnostics, Cat. #: MPEG) with the capture antibody specific to PEG backbone and the detection antibody to the terminal methoxy group of vaccine PEGylated lipid. This sandwich ELISA was conducted according to the manufacturer's instructions.

### Lymphocyte proliferative responses to spike protein

Spleens were obtained from the mice (6–8 weeks old) with maternal mRNA-1273 vaccination during pregnancy. Splenic lymphocytes were enriched by density gradient centrifugation and then cultured in triplicate each with $2 \times 10^5$ cells in 200 μL RPMI 1640 medium containing 10% fetal calf serum in 96-well plates. Responder lymphocytes were grown in medium only as background controls and stimulated with SARS-CoV-2 spike protein (1 μg/mL), third-party stimulator of BSA (100 ng/mL), or non-specific mitogen of Con-A (1 μg/mL). For the measurement of lymphocyte proliferation, day 5 cells were first subjected to 16-h incubation with tritiated thymidine (ICN Biomedicals) at a final concentration of 1 μCi per well and then harvested for counting incorporated tritium in a liquid scintillation counter (1450 Microbeta Plus counter). Lymphocyte proliferation was determined by the readout of incorporated tritium as counts per minute. Controls were the mice with maternal saline injection.

### IFN-γ and IL-2 ELISpot assay

Murine IFN-γ/IL-2-secreting T cells were quantified by mouse IFN-γ and IL-2 ELISpot Kits according to the manufacturer's instructions (R&D Systems). Briefly, splenic lymphocytes of each animal subject were enriched by density gradient centrifugation and then examined for their CD3 T cell fractions by flow cytometry after the treatment of fluorescence-conjugated anti-CD3 antibodies (BioLegend). Wells in the microplates were first rinsed with culture media of RPMI 1640 containing 10% fetal calf serum for 20 min at room temperature. Then, cells were loaded into wells in triplicate at a dose of $10^6$/100 μL culture media per well and incubated in a humidified 37°C $CO_2$ incubator for 2 days under the stimulation of SARS-CoV-2 spike protein (1 μg/mL, GTX02774-pro, GeneTex). The plates were washed with Wash Buffer four times and incubated with diluted detection antibody mixture (100 μL/well) for 2 h at room temperature on a rocking platform. After wash, the plates were incubated with diluted Streptavidin-AP Concentrate A (100 μL/well) for 2 h. The final wash was followed by the treatment of BCIP/NBT Substrate for 1 h. After the chromogen was decanted, the plates were washed with deionized water and dried at room temperature. Plates were scanned and counted on an immunospot analyzer (Cellular Technologies Ltd). The readouts of spike-reactive IFN-γ/IL-2-secreting T cells in each mouse were divided by its splenic $CD3^+$ T cell fraction to estimate their frequencies per million T cells.

### Neutralization assay with SARS-CoV-2 spike pseudovirus

HEK-293T cells stably expressing human ACE2 (293T-ACE2 cells) were grown in 96-well plates ($6 \times 10^4$ cells/well) at 37°C with 5% $CO_2$ for 24 h. Serial 2-fold dilutions of mouse sera were mixed with SARS-CoV-2 wild-type spike pseudotyped lentivirus containing luciferase gene (4,000 relative infection unit), provided by RNA Technology Plateform and Gene Manipulation Core, Academia Sinica, in DMEM with 1% fetal bovine serum (FBS) at 37°C for 1 h. Then, the serum-pseudovirus mixtures were added to 293T-ACE2 cells, which was incubated at 37°C with 5% $CO_2$ for 24 h and in DMEM with 10% FBS for another 24 h. The luciferase activity was determined by Bright-Glo Luciferase Assay kit (Promega, Madison, WI, USA) and the Synergy 2 (BioTek, Winooski, VT, USA) microplate reader.[39] Neutralization titer was determined by the highest serum dilution that reduced the viral infectivity by at least 50%, compared with the corresponding control wells without sera added.

### Histological examination of mRNA-LNPs and spike protein by immunofluorescence staining

The fetuses were fixed in 4% paraformaldehyde overnight and embedded in paraffin. Tissue sections were deparaffinized, rehydrated, and then subjected to heat-induced antigen retrieval. After permeabilization with Tween 20 and blocked with 1% BSA, the sections were incubated with primary antibodies against PEG (1:100, PEG-B-47, ab51257, Abcam) and spike protein (1:100, chimeric mAb, D001, MBS8119537) for 1.5 h, followed by fluorescence-conjugated donkey anti-rabbit IgG (1:100, Poly4064, BioLgend) and rat anti-human IgG Fc (1:100, M1310G05, BioLgend). Visualization of the nuclei was achieved by Hoechst 33342 staining (1:20,000, Invitrogen). Sections were mounted with Dako fluorescence mounting medium. Images were taken using a confocal microscope.

### Statistical analyses

All error bar charts were shown as 95% confidence intervals (boxed areas) for the means (transverse lines crossing the boxes) along with superimposed data points of individual mice. The equality of means was examined by Student's t test between two independent or paired groups, or by one-way analysis of variance (ANOVA)

among three or more groups with post hoc Fisher's least significant difference (LSD) multiple comparisons. Differences were regarded as significant in all tests at $p < 0.05$.

## DATA AND CODE AVAILABILITY

- Data reported in this paper will be shared by the correspondence author upon request.
- Any additional information required to reanalyze the data reported in this paper is available from the correspondence author upon request.

## ACKNOWLEDGMENTS

Special thanks go to Shiang-Chi Chen for her construction of graphic abstract. Shiang-Chi Chen is now studying for Master of Fine Arts (MFA) in the Visual Development, Academy of Art University, San Francisco, California, USA.

This project was financially supported by NSTC-112-2314-B-182-033 (J.-C.C.) and NSTC-113-2321-B-182-003 (C.-H.C.) from the National Science and Technology Council (NSTC), Taiwan, and CMRPG3N0931 (J.-C.C.) from Chang Gung Medical Foundation.

## AUTHOR CONTRIBUTIONS

J.-C.C. conceptualized this study, acquired funding, performed *in utero* injection, analyzed the data, prepared the figures and wrote the manuscript. M.-H.H. conducted the experiments of all immunoassays and RT-PCR. R.-L.K. and L.-T.W. performed pseudovirus neutralization assay. M.-L.K. helped to design the experiments of anti-spike $IgG_{2a}$ allotypes. L.-Y.T. and H.-L.C. performed immunostaining and assisted in experiments and animal surgery as well as care. C.-H.C. conceptualized this study, acquired funding, assisted in data analyses, supervised and coordinated the overall research, and edited the manuscript.

## DECLARATION OF INTERESTS

The authors declare no competing interests.

## SUPPLEMENTAL INFORMATION

Supplemental information can be found online at https://doi.org/10.1016/j.omtn.2025.102489.

## REFERENCES

1. Baden, L.R., El Sahly, H.M., Essink, B., Kotloff, K., Frey, S., Novak, R., Diemert, D., Spector, S.A., Rouphael, N., Creech, C.B., et al. (2021). Efficacy and Safety of the mRNA-1273 SARS-CoV-2 Vaccine. N. Engl. J. Med. *384*, 403–416.
2. Polack, F.P., Thomas, S.J., Kitchin, N., Absalon, J., Gurtman, A., Lockhart, S., Perez, J.L., Pérez Marc, G., Moreira, E.D., Zerbini, C., et al. (2020). Safety and Efficacy of the BNT162b2 mRNA Covid-19 Vaccine. N. Engl. J. Med. *383*, 2603–2615.
3. Qin, S., Tang, X., Chen, Y., Chen, K., Fan, N., Xiao, W., Zheng, Q., Li, G., Teng, Y., Wu, M., and Song, X. (2022). mRNA-based therapeutics: powerful and versatile tools to combat diseases. Signal Transduct. Targeted Ther. *7*, 166.
4. Gray, K.J., Bordt, E.A., Atyeo, C., Deriso, E., Akinwunmi, B., Young, N., Baez, A.M., Shook, L.L., Cvrk, D., James, K., et al. (2021). Coronavirus disease 2019 vaccine response in pregnant and lactating women: a cohort study. Am. J. Obstet. Gynecol. *225*, 303.e1–303.e17.
5. Jamieson, D.J., and Rasmussen, S.A. (2022). An update on COVID-19 and pregnancy. Am. J. Obstet. Gynecol. *226*, 177–186.
6. Halasa, N.B., Olson, S.M., Staat, M.A., Newhams, M.M., Price, A.M., Pannaraj, P.S., Boom, J.A., Sahni, L.C., Chiotos, K., Cameron, M.A., et al. (2022). Maternal Vaccination and Risk of Hospitalization for Covid-19 among Infants. N. Engl. J. Med. *387*, 109–119.
7. Rasmussen, S.A., and Jamieson, D.J. (2022). Covid-19 Vaccination during Pregnancy - Two for the Price of One. N. Engl. J. Med. *387*, 178–179.
8. Swingle, K.L., Safford, H.C., Geisler, H.C., Hamilton, A.G., Thatte, A.S., Billingsley, M.M., Joseph, R.A., Mrksich, K., Padilla, M.S., Ghalsasi, A.A., et al. (2023). Ionizable Lipid Nanoparticles for In Vivo mRNA Delivery to the Placenta during Pregnancy. J. Am. Chem. Soc. *145*, 4691–4706.
9. Santos, A., Sauer, M., Neil, A.J., Solomon, I.H., Hornick, J.L., Roberts, D.J., Quade, B.J., and Parra-Herran, C. (2022). Absence of SARS-CoV-2 Spike glycoprotein expression in placentas from individuals after mRNA SARS-CoV-2 vaccination. Mod. Pathol. *35*, 1175–1180.
10. Prahl, M., Golan, Y., Cassidy, A.G., Matsui, Y., Li, L., Alvarenga, B., Chen, H., Jigmeddagva, U., Lin, C.Y., Gonzalez, V.J., et al. (2022). Evaluation of transplacental transfer of mRNA vaccine products and functional antibodies during pregnancy and infancy. Nat. Commun. *13*, 4422.
11. (CHMP), C.f.M.P.f.H.U. (2021). Spikevax (Previously COVID-19 Vaccine Moderna) : EPAR - Public Assessment Report (European Medicines Agency). EMA/15689/2021.
12. Riley, R.S., Kashyap, M.V., Billingsley, M.M., White, B., Alameh, M.G., Bose, S.K., Zoltick, P.W., Li, H., Zhang, R., Cheng, A.Y., et al. (2021). Ionizable lipid nanoparticles for in utero mRNA delivery. Sci. Adv. *7*, eaba1028.
13. Pardi, N., Tuyishime, S., Muramatsu, H., Kariko, K., Mui, B.L., Tam, Y.K., Madden, T.D., Hope, M.J., and Weissman, D. (2015). Expression kinetics of nucleoside-modified mRNA delivered in lipid nanoparticles to mice by various routes. J. Contr. Release *217*, 345–351.
14. Etti, M., Calvert, A., Galiza, E., Lim, S., Khalil, A., Le Doare, K., and Heath, P.T. (2022). Maternal vaccination: a review of current evidence and recommendations. Am. J. Obstet. Gynecol. *226*, 459–474.
15. Snodgrass, H.R., Kisielow, P., Kiefer, M., Steinmetz, M., and von Boehmer, H. (1985). Ontogeny of the T-cell antigen receptor within the thymus. Nature *313*, 592–595.
16. Chen, J.C., Chan, C.C., Wu, C.J., Ou, L.S., Yu, H.Y., Chang, H.L., Tseng, L.Y., and Kuo, M.L. (2016). Fetal Phagocytes Take up Allergens to Initiate T-Helper Cell Type 2 Immunity and Facilitate Allergic Airway Responses. Am. J. Respir. Crit. Care Med. *194*, 934–947.
17. Chen, J.C., Ou, L.S., Kuo, M.L., Tseng, L.Y., and Chang, H.L. (2020). Fetal exposure to oncoantigen elicited antigen-specific adaptive immunity against tumorigenesis. J. Immunother. Cancer *8*, e000137.
18. Furukawa, S., Kuroda, Y., and Sugiyama, A. (2014). A comparison of the histological structure of the placenta in experimental animals. J. Toxicol. Pathol. *27*, 11–18.
19. Malassine, A., Frendo, J.L., and Evain-Brion, D. (2003). A comparison of placental development and endocrine functions between the human and mouse model. Hum. Reprod. Update *9*, 531–539.
20. Dilworth, M.R., and Sibley, C.P. (2013). Review: Transport across the placenta of mice and women. Placenta *34*, S34–S39.
21. Enders, A.C., and Carter, A.M. (2004). What can comparative studies of placental structure tell us?–A review. Placenta *25*, S3–S9.
22. Shimabukuro, T.T., Kim, S.Y., Myers, T.R., Moro, P.L., Oduyebo, T., Panagiotakopoulos, L., Marquez, P.L., Olson, C.K., Liu, R., Chang, K.T., et al. (2021). Preliminary Findings of mRNA Covid-19 Vaccine Safety in Pregnant Persons. N. Engl. J. Med. *384*, 2273–2282.
23. Zauche, L.H., Wallace, B., Smoots, A.N., Olson, C.K., Oduyebo, T., Kim, S.Y., Petersen, E.E., Ju, J., Beauregard, J., Wilcox, A.J., et al. (2021). Receipt of mRNA Covid-19 Vaccines and Risk of Spontaneous Abortion. N. Engl. J. Med. *385*, 1533–1535.
24. DeSilva, M., Haapala, J., Vazquez-Benitez, G., Vesco, K.K., Daley, M.F., Getahun, D., Zerbo, O., Naleway, A., Nelson, J.C., Williams, J.T.B., et al. (2022). Evaluation of Acute Adverse Events after Covid-19 Vaccination during Pregnancy. N. Engl. J. Med. *387*, 187–189.
25. Banoun, H. (2023). mRNA: Vaccine or Gene Therapy? The Safety Regulatory Issues. Int. J. Mol. Sci. *24*, 10514.
26. Zhang, L., Richards, A., Barrasa, M.I., Hughes, S.H., Young, R.A., and Jaenisch, R. (2021). Reverse-transcribed SARS-CoV-2 RNA can integrate into the genome of cultured human cells and can be expressed in patient-derived tissues. Proc. Natl. Acad. Sci. USA *118*, e2105968118.
27. Guerriaud, M., and Kohli, E. (2022). RNA-based drugs and regulation: Toward a necessary evolution of the definitions issued from the European union legislation. Front. Med. *9*, 1012497.
28. Roltgen, K., Nielsen, S.C.A., Silva, O., Younes, S.F., Zaslavsky, M., Costales, C., Yang, F., Wirz, O.F., Solis, D., Hoh, R.A., et al. (2022). Immune imprinting, breadth of

variant recognition, and germinal center response in human SARS-CoV-2 infection and vaccination. Cell *185*, 1025–1040.e1014.

29. Fertig, T.E., Chitoiu, L., Marta, D.S., Ionescu, V.S., Cismasiu, V.B., Radu, E., Angheluta, G., Dobre, M., Serbanescu, A., Hinescu, M.E., and Gherghiceanu, M. (2022). Vaccine mRNA Can Be Detected in Blood at 15 Days Post-Vaccination. Biomedicines *10*, 1538.

30. Ogata, A.F., Cheng, C.A., Desjardins, M., Senussi, Y., Sherman, A.C., Powell, M., Novack, L., Von, S., Li, X., Baden, L.R., and Walt, D.R. (2022). Circulating Severe Acute Respiratory Syndrome Coronavirus 2 (SARS-CoV-2) Vaccine Antigen Detected in the Plasma of mRNA-1273 Vaccine Recipients. Clin. Infect. Dis. *74*, 715–718.

31. Yonker, L.M., Swank, Z., Bartsch, Y.C., Burns, M.D., Kane, A., Boribong, B.P., Davis, J.P., Loiselle, M., Novak, T., Senussi, Y., et al. (2023). Circulating Spike Protein Detected in Post-COVID-19 mRNA Vaccine Myocarditis. Circulation *147*, 867–876.

32. Munisha, M., and Schimenti, J.C. (2021). Genome maintenance during embryogenesis. DNA Repair *106*, 103195.

33. Comaills, V., Kabeche, L., Morris, R., Buisson, R., Yu, M., Madden, M.W., LiCausi, J.A., Boukhali, M., Tajima, K., Pan, S., et al. (2016). Genomic Instability Is Induced by Persistent Proliferation of Cells Undergoing Epithelial-to-Mesenchymal Transition. Cell Rep. *17*, 2632–2647.

34. Mohrenweiser, H., and Zingg, B. (1995). Mosaicism: the embryo as a target for induction of mutations leading to cancer and genetic disease. Environ. Mol. Mutagen. *25 Suppl 26*, 21–29.

35. Bose, S.K., Menon, P., and Peranteau, W.H. (2021). In Utero Gene Therapy: Progress and Challenges. Trends Mol. Med. *27*, 728–730.

36. Trepotec, Z., Lichtenegger, E., Plank, C., Aneja, M.K., and Rudolph, C. (2019). Delivery of mRNA Therapeutics for the Treatment of Hepatic Diseases. Mol. Ther. *27*, 794–802.

37. Munnik, T., and Wierzchowiecka, M. (2013). Lipid-binding analysis using a fat blot assay. Methods Mol. Biol. *1009*, 253–259.

38. Golan, Y., Prahl, M., Cassidy, A., Lin, C.Y., Ahituv, N., Flaherman, V.J., and Gaw, S.L. (2021). Evaluation of Messenger RNA From COVID-19 BTN162b2 and mRNA-1273 Vaccines in Human Milk. JAMA Pediatr. *175*, 1069–1071.

39. Liu, K.T., Gong, Y.N., Huang, C.G., Huang, P.N., Yu, K.Y., Lee, H.C., Lee, S.C., Chiang, H.J., Kung, Y.A., Lin, Y.T., et al. (2022). Quantifying Neutralizing Antibodies in Patients with COVID-19 by a Two-Variable Generalized Additive Model. mSphere *7*, e0088321.

OMTN, Volume *36*

## Supplemental information

# mRNA-1273 is placenta-permeable and immunogenic in the fetus

**Jeng-Chang Chen, Mei-Hua Hsu, Rei-Lin Kuo, Li-Ting Wang, Ming-Ling Kuo, Li-Yun Tseng, Hsueh-Ling Chang, and Cheng-Hsun Chiu**

**A**

**Derivation of the mathematical formula for mRNA quantification**

In reverse transcription, **[cDNA] = [mRNA] x Yield (%)**

In quantitative PCR, **[DNA amplicons] = [cDNA] x $2^{Ct}$ = [mRNA] x Yield x $2^{Ct}$**

⇒ **[DNA amplicons] / Yield = [mRNA] x $2^{Ct}$**

Take natural logarithm (Ln) of both sides

**Ln([DNA amplicons] / Yield) = Ln([mRNA] x $2^{Ct}$)**

**= Ln[mRNA] + Ln($2^{Ct}$)**

**= Ln[mRNA] + Ct x Ln(2)**

**= Ln[mRNA] + Ct x 0.6931**

So **Ln[mRNA] = - 0.6931 x Ct + Ln([DNA amplicons] / Yield)**

In the case of common (decimal) logarithm, the formula will be:

**log[mRNA] = - 0.3010 x Ct + log([DNA amplicons] / Yield)**

- **[mRNA]** is the amount of sample mRNA that serves as the template to reversely transcribe cDNA, recorded as **[cDNA]**.
- **Yield** refers to reverse transcription efficiency for the reverse transcriptase used.
- **[DNA amplicons]** represents **[cDNA]** products after amplification for a period of **Ct**.
- **Ct** is the cycle threshold, as the thermal cycles required for the fluorescent signal to cross the threshold (corresponding to **[DNA amplicons]**) in the exponential phase of DNA amplification.

**B**

**3-fold serial dilutions of cDNA**

| Ct values | Moderna CmRNA (pg/uL) | Ln(CmRNA) |
|---|---|---|
| 6.4105 | 10,000.0000 | 9.2103 |
| 9.9689 | 3,333.3333 | 8.1117 |
| 14.7061 | 1,111.1111 | 7.0131 |
| 16.2043 | 370.3704 | 5.9145 |
| 17.8025 | 123.4568 | 4.8159 |
| 19.3628 | 41.1523 | 3.7173 |
| 21.0108 | 13.7174 | 2.6187 |
| 22.7968 | 4.5725 | 1.5201 |
| 24.7587 | 1.5242 | 0.4214 |
| 26.2916 | 0.5081 | -0.6772 |
| 27.6568 | 0.1694 | -1.7758 |
| 29.3245 | 0.0565 | -2.8744 |
| 31.0932 | 0.0188 | -3.9730 |
| 32.9435 | 0.0063 | -5.0716 |
| 34.8456 | 0.0021 | -6.1702 |

**3-fold serial dilutions of mRNA**

| Ct values | Moderna CmRNA (pg/uL) | Ln(CmRNA) |
|---|---|---|
| 5.3557 | 10,000.0000 | 9.2103 |
| 5.9440 | 3,333.3333 | 8.1117 |
| 10.8954 | 1,111.1111 | 7.0131 |
| 12.8743 | 370.3704 | 5.9145 |
| 14.9249 | 123.4568 | 4.8159 |
| 16.5962 | 41.1523 | 3.7173 |
| 17.9110 | 13.7174 | 2.6187 |
| 19.7502 | 4.5725 | 1.5201 |
| 21.6020 | 1.5242 | 0.4214 |
| 23.4505 | 0.5081 | -0.6772 |
| 24.7337 | 0.1694 | -1.7758 |
| 25.9041 | 0.0565 | -2.8744 |
| 25.8494 | 0.0188 | -3.9730 |
| 27.8912 | 0.0063 | -5.0716 |
| 26.6382 | 0.0021 | -6.1702 |

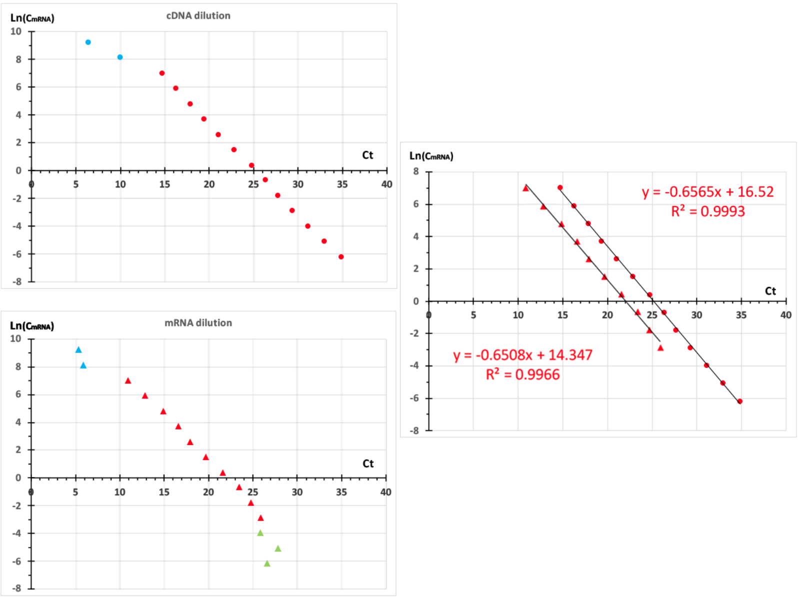


**C**

| Ct values | Moderna CmRNA (pg/uL) | Ln(CmRNA) |
|---|---|---|
| 5.6808 | 10,000.0000 | 9.2103 |
| 7.7765 | 3,333.3333 | 8.1117 |
| 12.5616 | 1,111.1111 | 7.0131 |
| 14.2327 | 370.3704 | 5.9145 |
| 15.8015 | 123.4568 | 4.8159 |
| 17.2555 | 41.1523 | 3.7173 |
| 19.1251 | 13.7174 | 2.6187 |
| 21.0317 | 4.5725 | 1.5201 |
| 22.7946 | 1.5242 | 0.4214 |
| 24.1883 | 0.5081 | -0.6772 |
| 25.7385 | 0.1694 | -1.7758 |
| 27.2565 | 0.0565 | -2.8744 |
| 28.6493 | 0.0188 | -3.9730 |
| 30.1717 | 0.0063 | -5.0716 |
| 32.0168 | 0.0021 | -6.1702 |
| 33.1922 | 0.0000 | Negative control |

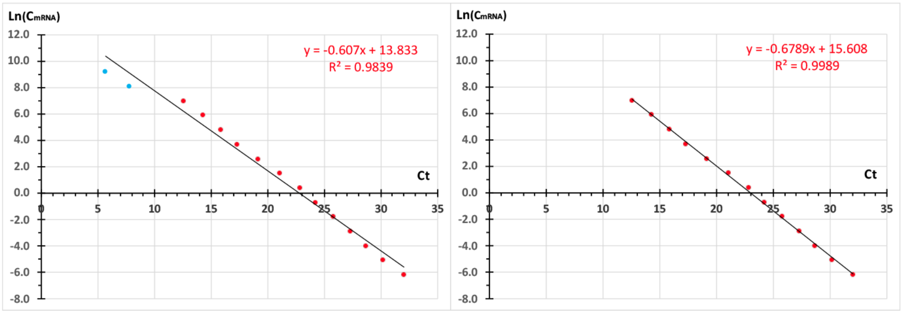


**Figure S1. Absolute quantification of spike mRNA by RT-PCR (A)** The formula for spike mRNA quantification was mathematically derived. In reverse transcription, cDNA transcripts are converted proportionally to input target mRNA amount,[S1,2] shown as [cDNA] = [mRNA] x Yield (%).[S3] Yield refers to reverse transcription efficiency for the reverse transcriptase used. In quantitative RT-PCR using cDNA reversely transcribed from mRNA, Ln[mRNA] is linearly related to Ct with a slope of -0.6931 and a vertical intercept of Ln([DNA amplicons] / Yield). **(B)** Serial dilution strategies of spike mRNA influenced the detection sensitivity of the spike mRNA standard curves. The standard curve of spike mRNA in RT-PCR was created in

reference to mRNA-1273, using 3-fold serial dilutions of cDNA reversely-transcribed from $10^4$ pg/μL mRNA-1273 (circular data points, upper panels) or cDNA reversely-transcribed individually from 3-fold serial dilutions of $10^4$ pg/μL mRNA-1273 (triangular data points, lower panels). The cDNA synthesized by the two dilution approaches was amplified by PCR run in duplicate on the same plate with the identical experimental condition, but analyzed separately to obtain the Ct value in correspondence to each spike mRNA concentration. In both approaches, the outliers (blue or green data points in dot plots) were masked to obtain the best fitting lines with $R^2$ of >0.99. The best fitting standard lines (X= Ct and Y= Ln($C_{mRNA}$)) were almost in parallel with nearly identical slopes (red data points, right panel). Their distinction lay in the vertical intercepts, which were mainly ascribed to a discrepancy in DNA amplicon amount corresponding to the Ct value preset by the software at data analyses. Apparently, the former (circular data points) had a better sensitivity in the quantifying spike mRNA of the unknowns than the latter (triangular data points), which showed the clear deviation of the final three mRNA dilutions (green triangular data points) away from the fitting line. In a given experimental condition, the reverse transcription yields of cDNA are in proportion to target mRNA template amounts.[S3] Once mRNA was serially diluted past a certain threshold, the rarity of target mRNA available in reverse transcription reactions significantly lowered the reverse transcription efficiency.[S2] It gave rise to cDNA of low abundance, which in turn heightened the Monte Carlo effect on PCR amplification due to the low probability of primer annealing to target cDNA.[S1] Under these circumstances, the amplified products failed to truly reflect the cDNA abundance, leading to the outlier Ct values in correspondence to the final 3 dilutions of mRNA-1273. **(C)** Absolute spike mRNA quantification in various tissues was conducted by RT-PCR using the standard curve method. The unknowns were compared to the standard curve so as to extrapolate a value. The standard curve was constructed by 3-fold serial dilutions of cDNA reversely-transcribed from $10^4$ pg/μL mRNA-1273 with corresponding spike mRNA of $10^4$ - 0.0012 pg/μL (left panel). On a scatter plot (middle panel), there was a linear relationship between natural logarithm of spike mRNA template amount ($C_{mRNA}$) and cycle threshold (Ct). The equation of the best fitting line was: $Ln(C_{mRNA}) = -0.6789\ Ct + 15.608$ (right panel) to the exclusion of two left upper data points (blue, outliers, middle panel) that did not fit the trend line very well. This dataset of a standard trend line was obtained from a representative experiment of RT- PCR to quantify spike mRNA in trunk soft tissues of the fetuses with maternal 4.0 μg mRNA-1273 vaccination. Ln($C_{mRNA}$) and their corresponding Ct values satisfied a linear relationship within the range of 0.0021 and 1111.1111 pg/μL mRNA-1273. Within this demarcated mRNA range (corresponding to Ct values between 32.0168 and 12.5616), samples' mRNA levels could be accurately determined via the linear equation by inputting the independent variables of Ct values.[S4] When a Ct value was equal to or higher than 33.1922 of negative controls (fetal soft tissues with gestational maternal saline injection), the sample was negative for spike mRNA. If a Ct value ranged between 32.0168 (corresponding to 0.0021 pg/μL spike mRNA) and 33.1922 (negative controls), the sample's mRNA level was recorded as <0.0021 pg/μL. The slope of −0.6789 in this equation was close to the theoretical slope of −0.6931.

**Supplemental Reference:**


S1. Bustin, S.A., and Nolan, T. (2004). Pitfalls of quantitative real-time reverse-transcription polymerase chain reaction. J Biomol Tech *15*, 155-166.

S2. Curry, J., McHale, C., and Smith, M.T. (2002). Low efficiency of the Moloney murine leukemia virus reverse transcriptase during reverse transcription of rare t(8;21) fusion gene transcripts. Biotechniques *32*, 768-775.

S3. Stahlberg, A., Kubista, M., and Pfaffl, M. (2004). Comparison of reverse transcriptases in gene expression analysis. Clin Chem *50*, 1678-1680.

S4. Nolan, T., Hands, R.E., and Bustin, S.A. (2006). Quantification of mRNA using real-time RT-PCR. Nat Protoc *1*, 1559-1582.

**Supplemental tables were shown in Excel format as follows:**

**Table S1:** Spike mRNA levels in offspring's blood, placenta, liver, trunk soft tissue and spleen after maternal vaccination with 4.0 μg mRNA-1273

**Table S2:** Spike mRNA levels in offspring's blood, placenta, liver, trunk soft tissue and spleen after maternal vaccination with 0.2 μg mRNA-1273

**Table S3:** Optic density of serum anti-spike $IgG_{2a}$ allotypes detected by anti-mouse Igh-1a/b, anti-mouse Igh-1a and anti-mouse Igh-1b after gestationally maternal vaccination of 4 μg mRNA-1273

**Table S4:** Optic density of serum anti-spike $IgG_{2a}$ allotypes detected by anti-mouse Igh-1a/b, anti-mouse Igh-1a and anti-mouse Igh-1b by 4 weeks postpartum after gestationally maternal vaccination of 0.2 μg mRNA-1273
